# Evaluation of lead equivalence of radiation protection apparatuses as a function of tube potential and spectral shaping filter

**DOI:** 10.1002/acm2.12768

**Published:** 2019-11-18

**Authors:** Areej Fawzi Aljabal, Richard Ryan Wargo, Pei‐Jan Paul Lin

**Affiliations:** ^1^ Division of Medical Physics Department of Radiation Oncology Virginia Commonwealth University Richmond VA USA; ^2^ Department of Radiology Virginia Commonwealth University Richmond VA USA

**Keywords:** lead and non‐lead protective apparatus, lead equivalence, lead foil tape, spectral shaping filter

## Abstract

**Purpose:**

This study aims to evaluate the lead equivalence (LE) of radiation protective apparatuses under various combinations of tube potentials and spectral shaping filter.

**Method:**

In this study, the commercially available 3M™ Lead Foil Tape 421, with nominal lead thickness of 0.1 mm, was employed to determine the LE of four different radiation protective apparatuses. The LE of protective apparatus was determined by utilizing the X‐ray transmission curves obtained with the lead foil tape at 60–120 kVp in combination with the spectral shaping filters of 0.1, 0.2, 0.3, 0.6, and 0.9 mmCu. The experimental setup and test method, for the transmission measurements with narrow beam geometry, was performed in accordance to ASTM Designation F2547‐18 Standards. All measurements were obtained using cardiovascular interventional angiography system.

**Results:**

A much larger discrepancies between the measured LE and stated (nominal) LE were observed at low tube potential (<70 kVp) for non‐lead protective apparatus. At higher tube potentials (>80 kVp) and thicker spectral shaping filters, the measured LE appears to be more consistent with the manufacturer specified nominal thickness for the protective apparatus investigated. On the other hand, for the lead protective eyeglasses, the measured lead equivalence of both the lead side shield and the lens of eyeglasses (0.38 and 0.85 mmPb respectively) are consistent across all tube voltage.

**Conclusion:**

The conventional specification of LE without considering spectral shaping filter is a valid measure for tube voltages at and above 80 kVp. The measured LE generally exceed the specifications. The difference is most significant at lower tube potentials, and especially with thicker spectral shaping filters. At higher voltages (>100 kVp), the measured LE and the nominal LE are in good agreement with each other irrespective of the spectral shaping filter thickness.

## 1 INTRODUCTION

Conventional radiation protective apparatus (RPA) such as aprons and garments are made of lead or lead composite materials. Lead composite shielding materials have overcome the weight burden with lighter elements such as bismuth oxide and provide comparable protection and attenuation properties to lead at a certain X‐ray energy.^1^, ^2^, ^3^, ^4^ The composition of these materials is often not disclosed while only their lead equivalence (LE) in millimeter of lead (mmPb) is specified.^5^ The effectiveness of these non‐lead protective garments may be evaluated with attenuation measurement, and in terms of lead equivalency.^4^ In fact, the LE is energy dependent; therefore, they would only have the same X‐ray attenuation as lead at certain X‐ray energies. However, the transmitted spectra are quite different.^3^, ^5^ Although attenuation properties and effectiveness of lead and radiation protective garments have been studied by various investigators, the influence of spectral shaping filters (SSF) was not included in their investigations.^3^, ^5^, ^6^, ^7^, ^8^

The method to determine attenuation properties of shielding materials has been described by two international radiation standards. They are (a) International Electrotechnical Commission (IEC) 61331‐1,^9^ Protective Devices against Diagnostic Medical X‐radiation, Part 1: Determination of Attenuation Properties of Materials.^10^ (b) American Society for Testing and Materials (ASTM) F2547‐18, Standard Test Method for Determining the Attenuation in a Primary X‐ray Beam of Materials Used to Protect against Radiation Generated during the use of X‐ray Equipment.^11^ Both IEC and ASTM have established a standardized test method and experimental setup to measure the attenuation values of the protective materials in the primary and scattered X‐ray beam. The major differences between these two methods are in experimental setups. IEC standard provides three different experimental setups for attenuation ratio measurements: broad beam, inverse broad beam, and narrow beam geometries. Broad beam and inverse broad beam are defined as the standard test methods to determine LE value accounting for both primary and scattered radiation. Narrow beam geometry is defined as the standard test method to determine LE value accounting for primary radiation only. Alternatively, ASTM International has established two standard test methods ASTM F2547‐18 and ASTM F3094‐14. The ASTM F2547‐18 standard measures the attenuation values of the protective materials in the primary X‐ray beam under condition of narrow geometry. The ASTM F3094‐14 standard determines protection provided by X‐ray shielding materials from “Sources of Scattered X‐ray” using inverse broad‐beam geometry.

IEC 61331‐1 standard provides specification of LE only for garments that contain lead and at certain beam quality. In addition, due to setup difficulties within the IEC specification, one might not be able to comply with the requirements. Whereas the ASTM F2547‐18 Standard is a test method to measure the attenuation of the shielding materials in the range of 60–130 kVp tube potential. A study by Jones et al.^8^ followed both IEC and ASTM standardized methods to determine LE values of lead and non‐lead protective garments. The study showed that the lead‐based garment has the same nominal LE value at all kVp values, whereas the non‐lead garment’s LE value varies as a function of beam quality. The study stated that accurate measurement of scattered beams under broad‐beam geometry was challenging while primary beam measurement under narrow beam condition was much more useful. A more recent study^12^ showed that the LE value for shielding materials is influenced by both method of measuring the attenuation and beam quality.

The aim of this study is to determine the LE of radiation protective apparatus (RPA) due to the influence of varying tube potential and in combination with the SSFs. Measurement of LE was performed following ASTM F2547‐18 standard using the primary X‐ray beam under condition of narrow beam geometry.

## 2 MATERIALS AND METHODS

### 2.1 Measurement equipment

All measurements were conducted using cardiovascular interventional angiography (CIA) equipment. The CIA system was operated under the service mode so that the tube potential and SSFs can be selected manually. The primary radiation transmission measurements were obtained using Black Piranha Model 657 dosimeter system (RTI, Towaco, NJ 07082, USA). The acquisition parameters were large focus of 1.0 mm, tube current of 400 mA, pulse width of 100 ms, and tube potential between 60 and 120 kVp in increment of 10 kVp, in combination with SSFs of 0, 0.1, 0.2, 0.3, 0.6, and 0.9 mm of copper (mmCu).

### 2.2 Lead foil sheets and transmission curves

Typically, protective garments have nominal LE thickness of 0.25–0.5 mm (mmPb). The thinnest commercially available lead sheet, we were able to find, is 3M™ lead foil tape 421 (3M™ Corporate, St. Paul, MN) with a total nominal thickness of 6.3 mil (0.16 mm). As specified in the product brochure, it consists of 4.0 mil (0.10 mm) lead foil as the backing material and 2.3 mil (0.06 mm) as adhesive material. The 3M™ lead foil tape 421(LFT), here after 3M™ LFT, has been internally verified through attenuation measurements. It was found that the 3M™ LFT is, indeed, made of 0.1 mm lead foil. The transmission curves from this internal investigation were utilized to determine the LE of RPA.

### 2.3 Radiation protective apparatus (RPA)

Listed in Table 1 are four RPA investigated in this study. They are as follows: (a) surgical drape (non‐lead), (b) surgical cap (non‐lead), (c) thyroid shield (non‐lead), and (d) lead eyeglasses (lead lenses and lead side shield with vinyl sheet).

**Table 1:** Radiation protective apparatus (RPA) description and specification.

Testing protective apparatus	Nominal (mmPb)	Material type	Specification listed	Manufactures brand	Model
Drape	0.25	Lead free	Lead equivalence attenuated up to 98% at 80 kVp	AADCO Medical, Inc., Randolph, VT	X‐drape® sterile field X‐ray shielding drapes
Thyroid shield	0.50	Lead free	Not specified	Burlington Medical, Newport News, VA	Enviro‐lite™
Surgical cap	0.125	Lead free	Not specified	AADCO Medical, Inc., Randolph, VT	Disposable comfort‐fit™ X‐ray shielded procedure cap
Eyeglasses lens	0.75	Lead	Lead equivalence value measured at 150 kVp	(Burlington Medical, Newport News, VA).	ES40S lead eyeglasses
Side shield	0.35				

### 2.4 Experimental setup for X‐ray transmission measurements

**Figure 1:**
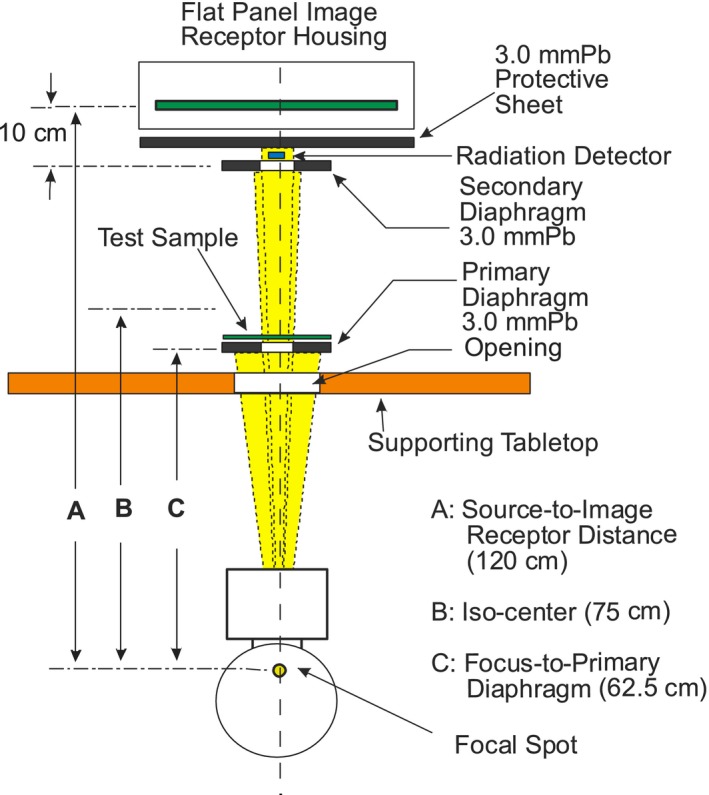
Experimental setup for measurement of X‐ray transmission, narrow beam geometry with slight adjustment to accommodate the interventional angiography system.

The experimental setup and test method for transmission measurements under narrow beam geometry were conducted in accordance to ASTM Designation F2547‐18 standards. The geometry of the measurement was maintained constant for all lead sheets and protective apparatus to insure consistency and reliability. The primary beam was measured with and without the shielding material (Test Sample) as shown in Fig. 1.

The transmission curves of 3M™ LFT were obtained under the following conditions: (a) tube potential in the range of 60–120 kVp, in increments of 10 kVp, and in combination with, (b) the SSFs of 0.0, 0.1, 0.2, 0.3, 0.6, and 0.9 mm copper (mmCu). In this study, the transmission ratio of the RPA was measured under the same measurement conditions described above. The transmission ratio of the RPA was superimposed onto the transmission curves of 3M™ LFT to determine the LE values of the RPAs. The LE was determined (calculated) by simple linear interpolation of the transmission data through 3M™ LFT, since we have 0.1 mmPb increment available for testing, that was based on the internal study. Then, the calculated LE values of RPA were compared with the nominal values listed on their labels across all beam qualities.

## 3 RESULTS

**Figure 2:**
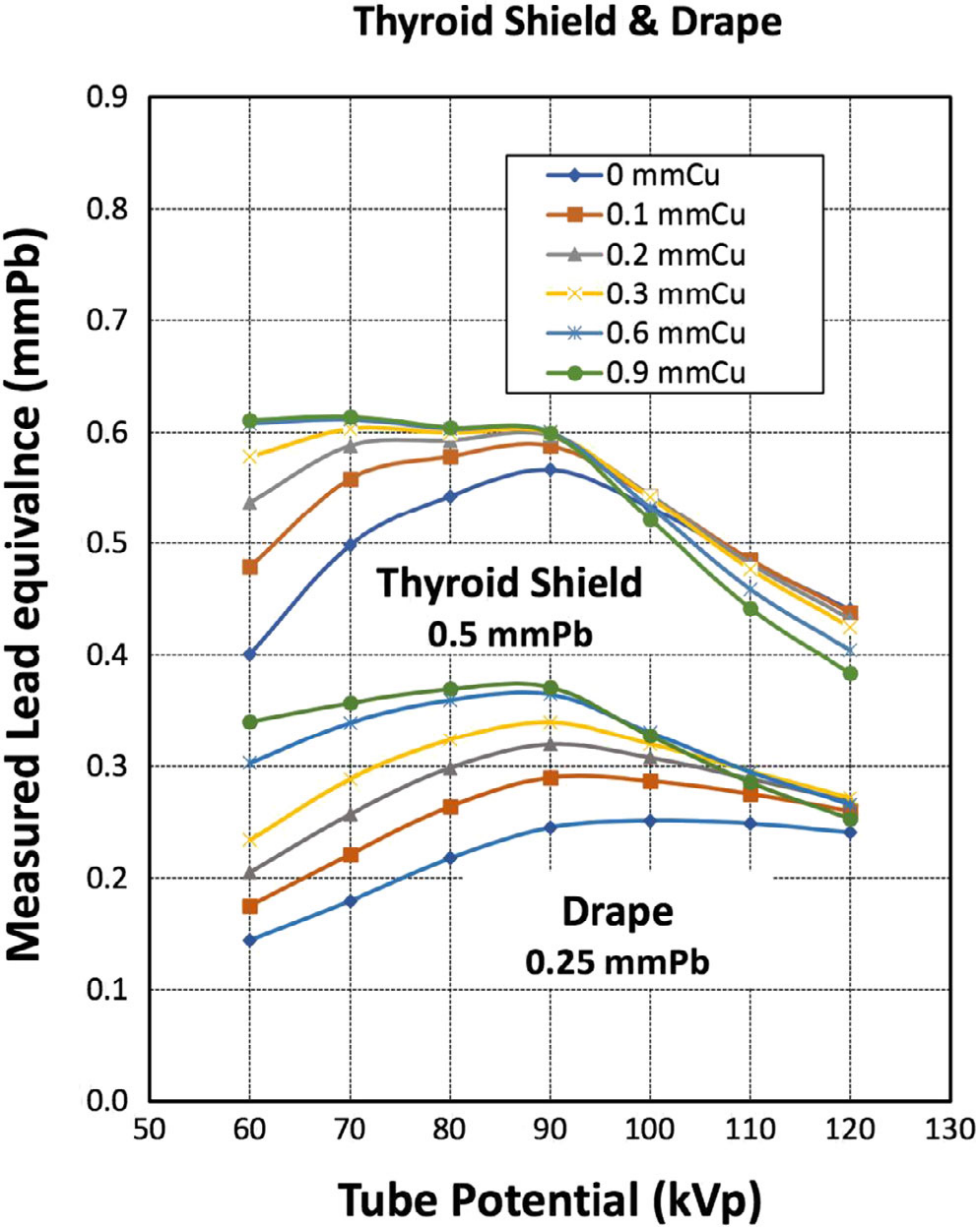
The measured lead equivalence (LE) in “mmPb” for thyroid shield and drape. The LEs of thyroid shield and the surgical drape vs X‐ray tube potential. Each curve represents different spectral shaping filters (mmCu). The thickness indicated in the figure is the nominal LE of the radiation protective apparatus.

**Figure 3:**
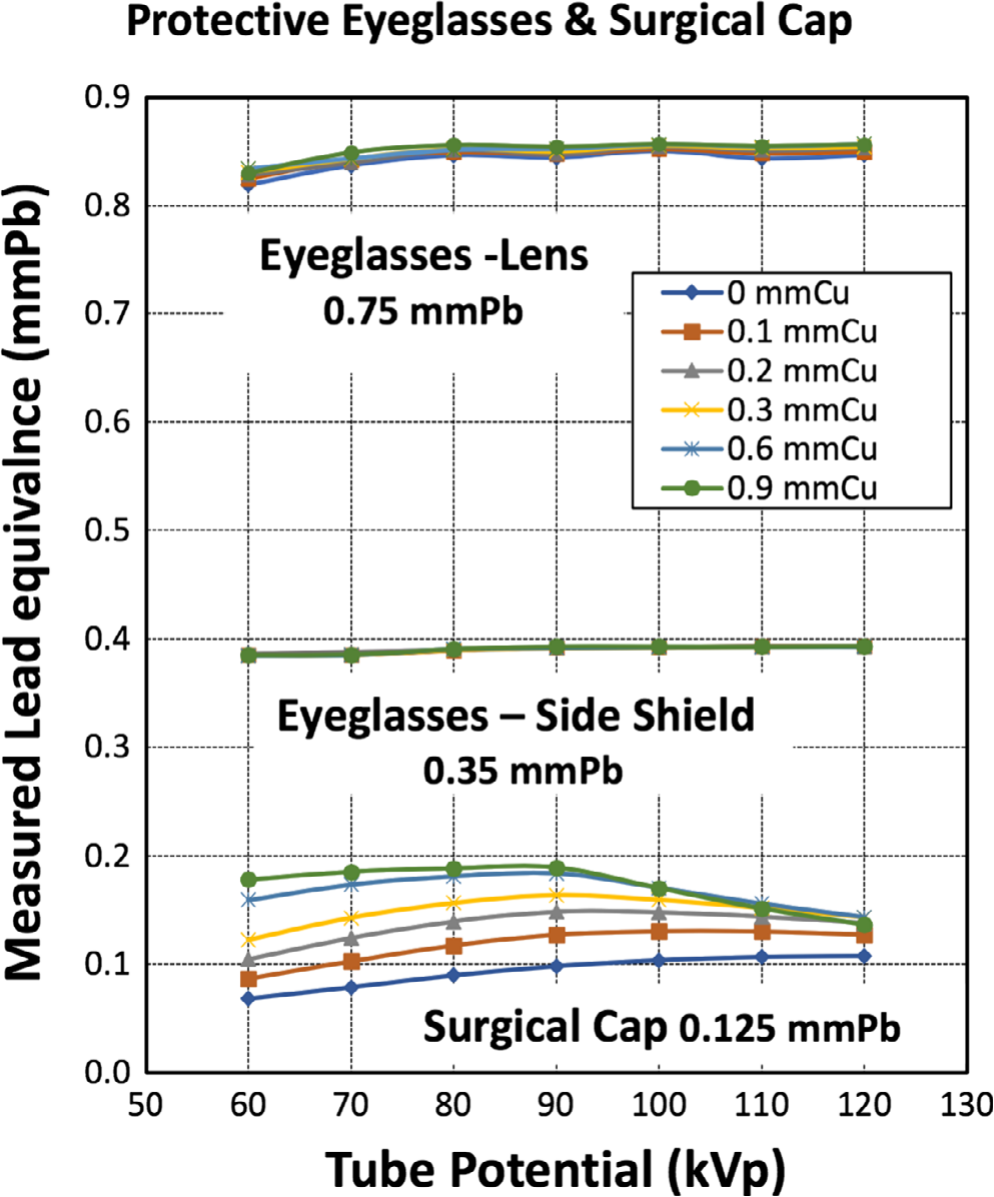
The measured lead equivalence (LE) in “mmPb” for the lens of eyeglasses, the side shield, and the surgical cap. The LEs of the lens of eyeglasses, the side shield, and the surgical cap vs X‐ray tube potential. Each curve represents different spectral shaping filters (mmCu). The thickness indicated in the figure is the nominal LE of the radiation protective apparatus.

The results are represented in Figures 2 and 3, and Table 2. The experiment and the LE measurements were performed for the diagnostic X‐ray energy range, 60–120 kVp, and in combination with different thicknesses of spectral shaping copper filters (0, 0.1, 0.2, 0.3, 0.6, and 0.9 mm). For demonstration, calculated LE values at selected tube potential (kVp) and the SSF (mmCu) are presented in Table 2. Illustrated in Fig. 2 is the measured LE values for *Thyroid shielding* and *Drape* investigated (both are lead‐free material) as a function of tube potential with SSF. Similarly, depicted in Figs. 3 is the measured LE values for the lens of the Eyeglasses and its side shielding (both are made of lead) and Surgical Cap (lead‐free) as a function of tube potential with SSFs.

**Table 2:** Calculated Lead equivalence of protective apparatus in millimeters of lead (mmPb) at 60, 80,100, and 120 kVp and with different spectral shaping filter mmCu.

Testing materials	60 kV at mmCu						80 kVp at mmCu						100 kVp at mmCu						120 kVp at mmCu					
	0	0.1	0.2	0.3	0.6	0.9	0	0.1	0.2	0.3	0.6	0.9	0	0.1	0.2	0.3	0.6	0.9	0	0.1	0.2	0.3	0.6	0.9
Drape	0.145	0.175	0.205	0.234	0.303	0.340	0.218	0.264	0.299	0.324	0.360	0.370	0.251	0.287	0.308	0.320	0.331	0.328	0.241	0.260	0.269	0.272	0.266	0.253
Thyroid shield	0.401	0.479	0.537	0.578	0.608	0.611	0.543	0.578	0.593	0.599	0.603	0.604	0.533	0.542	0.544	0.542	0.533	0.522	0.441	0.438	0.432	0.425	0.404	0.384
Surgical cap	0.068	0.087	0.105	0.123	0.159	0.178	0.090	0.117	0.139	0.156	0.181	0.189	0.104	0.130	0.148	0.159	0.170	0.170	0.108	0.127	0.138	0.143	0.143	0.137
Lens of eyeglasses	0.820	0.825	0.828	0.829	0.834	0.830	0.847	0.850	0.851	0.852	0.852	0.856	0.851	0.852	0.855	0.856	0.857	0.857	0.847	0.850	0.853	0.855	0.857	0.857
Side shield of eyeglasses	0.386	0.385	0.386	0.385	0.384	0.385	0.389	0.389	0.390	0.390	0.390	0.390	0.392	0.392	0.392	0.392	0.392	0.393	0.393	0.393	0.393	0.393	0.393	0.393

Table 2 shows the calculated LE “in mmPb” of RPAs investigated in this study, and the effect of different thicknesses of SSF (0, 0.1, 0.2, 0.3, 0.6, and 0.9 mmCu) at 60, 80, 100, and 120 kVp. Note, the same data at 70, 90, and 110 kVp were collected but was not included for presentation simplicity. In Table 2, the tube voltages of 60, 80, 100, and 120 kVp were chosen, since most of the LEs of RPA are specified and listed on their labels as the nominal LE at 80 or 120 kVp, but no SSF is considered.

## 4 DISCUSSION

### 4.1 Surgical drape

The measured LE, at 80 kVp and 0 mmCu, of the drape was approximately 0.22 mmPb. The LE was less than the specified value of 0.25 mmPb by 12%. At tube voltage higher than 80 kVp and with thicker SSF, the measured LE tends to be closer to, or higher than, the specified value. At tube voltages lower than 80 kVp and SSF thickness of <0.2 mmCu, the measured LE was less than the specified and increases with increasing SSF. The measured LE at 120 kVp corresponding to filter thickness of 0 and 0.9 mmCu were 0.24 and 0.25 mmPb, respectively. The measured LE was less than that specified by the manufacturer at 60 and 70 kVp and 0 mmCu, but tends to be closer to the specified values as SSF thickness is increased.

### 4.2 Thyroid shield

The measured LE, at 80 kVp and 0 mmCu, was 0.54 mmPb, which is 8% higher than the specified LE of 0.50 mmPb. As tube voltage is increased to higher than 100 kVp, the measured LE of the thyroid shield decreased to below the specified LE of 0.5 mmPb. For instance, the LE of the thyroid shield at 110 and 120 kVp with SSF of 0.0 mmCu were 0.48 and 0.44 mmPb, respectively.

### 4.3 Surgical cap

The LE value, at 80 kVp and 0 mmCu, of the surgical cap was 0.09 mmPb, approximately 28% less than the specified 0.125 mmPb. Without SSF in the beam, at tube voltage higher than 90 kVp, the LE tended to be closer to the specified value. For instance, surgical cap’s LE at 90, 100, 110, 120 kVp, and 0.0 mmCu were 0.098, 0.104, 0.107, and 0.108 mmPb respectively. Across all tube voltage, the LE values increased than specified value as SSF thickness increased.

### 4.4 Lens and side shield of protective eyeglasses

For the lead protective eyeglasses, the LE value of the lens was 0.85 mmPb, 13.3% higher than the “specified 0.75 mmPb.” While the measured LE for the side shield, at 80 kVp and 0 mmCu, was 0.38 mmPb, 8.6% higher than “specified 0.35 mmPb.” The LE values of both the lens and the side shield are relatively consistent over entire tube potential range and the SSFs; namely, 0.84 ± 0.011 mmPb and 0.39 ± 0.003 mmPb, respectively.

In the case of the lead RPA (4‐D) and Fig. 3, it is uniformly more effective in protection against radiation over the full range of radiation beam quality in this study, where “the full range of radiation beam quality” refers to the beam qualities in the combinations of the tube potentials and the SSFs. The lead RPAs’ LE is not adversely affected whether SSF is employed or not, since the “normalization” in obtaining the LE is also based on lead. Thus, the lead protective materials showed perfect consistency among all RPA investigated.

On the other hand, for the non‐lead RPA (4‐A and 4‐B), the LE varies substantially below 90 kVp by more than ±0.1 mmPb for the thyroid shield and the surgical drape shown in Fig. 2. For the surgical cap (4‐C), in Fig. 3, the LE varies by approximately ±0.06 mmPb.

The variation in the LE for non‐lead RPAs can be attributed to two reasons: (a) Since photoelectric effect is the dominant radiation interaction in the diagnostic X‐ray energies range and (b) as mentioned earlier, these protective apparatuses are made of composite materials with different K‐absorption energies.

The thyroid shield, for example, consists mainly of bismuth–antimony BiSb, which has X‐ray absorption edge energies of 90.52 and 30.49 keV, respectively.^13^ Thus, for lower X‐ray energies, 60 and 70 kVp, the effect of K‐edge is significant. The measured LE is optimum at tube potentials of 80 and 90 kVp and decreased at beam qualities >90 kVp regardless of the SSF thickness (mmCu).

Therefore, the non‐lead thyroid shield is effective at 80–90k kVp but less effective at the lower tube potentials below 80 kVp. At the higher tube potential and higher SSFs, the LE is close to the nominal values but is somewhat less effective. Although we do not know the exact composition of the surgical drape, LE of this non‐lead RPA has similar transmission properties as the thyroid shield. The surgical drape is also less effective at low tube potential (<80 kVp). The optimal protection is afforded at or around 80–90 kVp. Surgical cap also has less LE at low kVp with SSF < 0.3 mmCu which is less effective in radiation protection at low kVps. This proves that whatever the materials that non‐lead protective apparatuses are made of, these RPA are designed to curtail the radiation between 80 and100 kVp.

## 5 CONCLUSION

Our study showed how effective the lead and non‐lead RPAs would be in a clinical environment. Typically, the non‐lead RPA would not be as effective as it is claimed by the manufacturer for environment where the routine fluoroscopic tube potential is <80 to 90 kVp. Although the traditional specification of LE at 80 kVp with beam quality of HVL at 3 mmAl, without taking SSF into account, it is still a valid measure to specify the nominal LE value. One cannot expect the LE is valid for entire range of tube potentials encountered in diagnostic radiology. It is strongly suggested that even if the manufacturers do provide the LE values for RPA based on the traditional, conventional specification, it is advisable to evaluate the “true” LE under the clinical conditions the RPA is intended for. In addition, in the case when the LE cannot be identified through its label or appropriate document, one should first check the LE at 80 kVp with the beam quality of 3 mmAl HVL.

## CONFLICT OF INTEREST

The authors have no conflict of interest to declare.
